# Organizing Sequential Memory in a Neuromorphic Device Using Dynamic Neural Fields

**DOI:** 10.3389/fnins.2018.00717

**Published:** 2018-11-13

**Authors:** Raphaela Kreiser, Dora Aathmani, Ning Qiao, Giacomo Indiveri, Yulia Sandamirskaya

**Affiliations:** ^1^Institute of Neuroinformatics, University of Zurich and ETH Zurich, Zurich, Switzerland; ^2^The School of Electrical and Computer Engineering, Georgia Institute of Technology, Atlanta, GA, United States

**Keywords:** neuromorphic engineering, on-chip learning, sequence learning, dynamic neural fields, synaptic plasticity, neurorobotics, winner take all

## Abstract

Neuromorphic Very Large Scale Integration (VLSI) devices emulate the activation dynamics of biological neuronal networks using either mixed-signal analog/digital or purely digital electronic circuits. Using analog circuits in silicon to physically emulate the functionality of biological neurons and synapses enables faithful modeling of neural and synaptic dynamics at ultra low power consumption in real-time, and thus may serve as computational substrate for a new generation of efficient neural controllers for artificial intelligent systems. Although one of the main advantages of neural networks is their ability to perform on-line learning, only a small number of neuromorphic hardware devices implement this feature on-chip. In this work, we use a reconfigurable on-line learning spiking (ROLLS) neuromorphic processor chip to build a neuronal architecture for sequence learning. The proposed neuronal architecture uses the attractor properties of winner-takes-all (WTA) dynamics to cope with mismatch and noise in the ROLLS analog computing elements, and it uses its on-chip plasticity features to store sequences of states. We demonstrate, with a proof-of-concept feasibility study how this architecture can store, replay, and update sequences of states, induced by external inputs. Controlled by the attractor dynamics and an explicit destabilizing signal, the items in a sequence can last for varying amounts of time and thus reliable sequence learning and replay can be robustly implemented in a real sensorimotor system.

## 1. Introduction

Mixed-signal analog-digital neuromorphic Very Large Scale Integration (VLSI) systems emulate the biophysics of cortical neurons and synaptic connections between them using the physics of silicon electronic devices (Moradi et al., 2018). Computation and memory are co-localized in these systems. Furthermore, as communication of signals across neurons and modules is asynchronous and data-driven, this leads to ultra low-power consumption and highly efficient real-time processing. While some recent neuromorphic hardware devices aim at speeding up the processing time of computational neuroscience simulations, e.g., SpiNNaker (Furber et al., 2012) or HICANN (Schemmel et al., 2010; Benjamin et al., 2014), other neuromorphic hardware systems have been developed as basic research tools for emulating the properties of real cortical circuits in real-time (Chicca et al., 2014; Qiao et al., 2015). These latter systems are particularly well suited also as a neural computing substrate for real-time technical applications that can profit from their massively parallel architecture, ultra-low power consumption, and small form-factor, for example, in control of real-time robotic systems with embedded processing (Ijspeert, 2008; Conradt et al., 2009; Xi, 2016).

Several mixed signal analog/digital neuromorphic devices were previously used for spiking pattern classification (Mitra et al., 2009; Corradi and Indiveri, 2015; Kreiser et al., 2017), motor controllers for robotic devices (Serrano-Gotarredona, 2009; Perez-Peña et al., 2013; Perez-Pena et al., 2014b; Glatz et al., 2018; Kreiser et al., 2018a,b), simple stimulus-response based agents (Indiveri et al., 2001; Conradt et al., 2009), and were successful at the level of early sensory processing in vision and audition (Liu and Delbruck, 2010). Typically the neural network connectivity is determined at design-time off-line, to fulfill specific task requirements. However, neuromorphic systems with on-chip learning abilities (Mitra et al., 2009; Qiao et al., 2015; Davies et al., 2018). In particular, neuromorphic systems that have the ability to modify synaptic weights between neurons with biologically plausible plasticity mechanism allow the construction of low-power adaptive neural processing systems that can be used for building autonomous cognitive agents (Chicca et al., 2014).

In this work, we use a neuromorphic device that is equipped with analog on-chip learning circuits, in order to store a sequence of visual inputs in a robotic sensorimotor loop. Learning such sequence is enabled by a neural architecture that can cope with the challenges brought about by the mixed signal analog/digital neuromorphic hardware.

One of the main challenges in applications of mixed signal neuromorphic systems is that of devices mismatch—variability in properties of computing elements due to the fabrication process and the sub-threshold operation. This leads to output noise and variability in properties of neurons and synapses, if realized with analog circuits (Neftci et al., 2011). To make neuromorphic hardware work reliably in face of its variability, the silicon neural networks need to form representations that are stable against mismatch. Biological neural networks face a similar problem of fluctuations of biochemical parameters, nevertheless animals are capable of precise and reproducible behavior, thus biology must have developed efficient solutions to this problem.

One of these solutions is population dynamics with soft winner-take-all (WTA) connectivity. The WTA is a computational primitive that leads to continuous-attractor dynamics that were found to be characteristic for many cortical and subcortical neural networks (Wilson and Cowan, 1973; Gerstner and Kistler, 2002). Moreover, in the framework of Dynamic Neural Fields (DNFs), an analogy is drawn between population dynamics with WTA connectivity and behavioral dynamics, observed in experiments studying perceptual, motor, and cognitive behavior (Schöner and Spencer, 2015). DNFs, originally developed to describe the activation dynamics of large neural populations (Wilson and Cowan, 1973; Amari, 1977; Grossberg, 1988), have been used to account for human cognition (Johnson et al., 2008; Schöner and Spencer, 2015) and to develop cognitive architectures for robotics (Gaussier and Zrehen, 1994; Lipinski et al., 2009; Bicho et al., 2012; Richter et al., 2012; Sandamirskaya et al., 2013).

Robotic demonstrators are a novel tool entering the area of neuroscience that allows better understanding of brain structure and functionality by offering an “embodiment” for computational models (Thelen, 1995; Wolfgang and Jean-pierre, 2003; Pfeifer et al., 2007). A full spectrum of the currently ongoing projects in the area of neuromorphic robotics was listed and extensively analyzed in Krichmar and Wagatsuma (2011). The emerging tendency in brain-inspired robotics is to mimic the organization of nervous infrastructure in order to obtain similar functionality.

WTA connectivity is one of the organization schemes that was postulated in the past as an elementary computing unit for both neural processing and neuromorphic architectures (Indiveri et al., 2009; Rutishauser and Douglas, 2009; Neftci et al., 2013). DNF dynamics have been implemented on analog neuromorphic devices (Sandamirskaya, 2014) and in this paper, we build on this work to demonstrate how sequences of states, realized by application of WTA networks, can be learned in plastic synapses on a neuromorphic chip. The neuromorphic chip can be embedded in a robotic agent where states of the sequence are driven by sensory input. In particular, we show how on-chip plastic synapses store a sequence of visual information that is perceived by a neuromorphic camera (Delbruck and Lichtsteiner, 2006), mounted on a robotic agent.

Neuronal mechanisms for representing sequences have been studied both in humans (e.g., in behavioral experiments on learning movement sequences (Hikosaka et al., 2002; Deroost et al., 2006) or in serial order errors, e.g., in typing or language production (Henson, 1998), as well as in animals [e.g., in rats performing grooming movements (Aldridge and Berridge, 2003) or during navigation in mazes (Foster and Wilson, 2006)]. Furthermore, recent recordings from basal ganglia in humans allowed to gain insights into the neuronal mechanisms of learning movement sequences, in particular, in detecting errors in serially ordered sequences of tones (Herrojo Ruiz et al., 2014).

From a multitude of neural models for sequence learning (e.g., Deco and Rolls, 2005; Wörgötter and Porr, 2005; Rabinovich et al., 2006), we selected one that allows an agent to learn sequences from real sensory data and produce sequences through a physical motor system. It achieves the required stability and robustness of actuators plans and perceptual decisions using the DNF, or likewise WTA attractor dynamics, and its transition ability through an explicit destabilizing signal (Sandamirskaya and Schöner, 2010a). This model is inspired by neuronal findings on serial order encoding in the brain (Aldridge and Berridge, 1998; Clower and Alexander, 1998; Carpenter, 1999; Procyk et al., 2000) and behavioral data on serial order errors (Henson, 1998).

In this work, we implement the model on the ROLLS (Reconfigurable On-Line Learning Spiking) neuromorphic processor (Qiao et al., 2015). The model is a neural architecture that creates stable attractor states in a population of “ordinal” neurons that represent the ordinal position in a sequence. An active group of ordinal is associated—through synaptic plasticity—with a neuronal representation of the “content” for each item in a sequence. This content is represented with a population that features WTA network topology and is driven by sensory inputs during sequence learning. During learning, associations between ordinal positions and different regions on the content WTA are established. Learning is supported by co-existence of the attractor states in the two neural populations that are sustained for macroscopic amounts of time. During sequence replay—i.e., acting out of a memorized sequence,—each state in the content WTA is activated by the ordinal nodes for a variable amount of time, that is controlled by an external “condition of satisfaction” signal. To our knowledge, this simple example is the first demonstration of on-chip learning that proceeds autonomously in a mixed signal neuromorphic hardware in a closed sensorimotor loop.

## 2. Material and methods

### 2.1. Neuromorphic hardware: the ROLLS device

**Figure 1:**
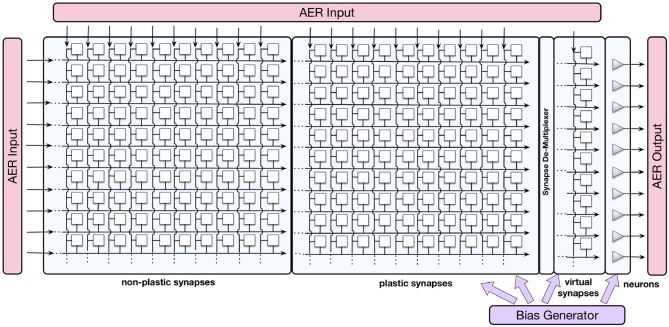
Block diagram of the ROLLS chip architecture. Triangles on the right represent silicon neurons, squares are synapses that are organized in three arrays: non-plastic, plastic, and virtual. The AER blocks manage the input and output traffic of spikes, and the bias generator allows to program different parameter settings of the analog circuits.

The ROLLS neuromorphic device used in this work comprises mixed-signal analog/digital neuron and synapse circuits that can exhibit a range of biologically realistic dynamics (refractory period, time-course of integration and leakage, firing rate adaptation, short- and long-term plasticity, etc.). The silicon neuron circuits implement a model of the adaptive exponential integrate-and-fire (IF) neuron (Brette et al., 2007). A schematic diagram of the chip architecture is shown in Figure 1.

The device comprises 256 analog silicon neuron circuits, an array of 256 × 256 non-plastic programmable synapses, an array of 256 × 256 plastic synapses, and a 256 × 2 linear integrator filters—“virtual synapses” that can be used to direct external inputs to the neurons. The non-plastic synapses consist of analog circuits that reproduce short-term adaptation dynamics (Rasche and Hahnloser, 2001; Boegerhausen et al., 2003) and digital circuits that set the programmable weights. On ROLLS, four different weights are available on chip and each one of them can be set to a required value regulated by on-chip biases.

The plastic synapses contain analog learning circuits and digital state-holding logic. The learning circuits implement the synaptic plasticity model proposed in Brader et al. (2007), which is particularly well-suited to VLSI implementation. According to this rule, the synaptic weights are updated based on the timing of the pre-synaptic spike, the state of the post-synaptic neuron‘s membrane potential, and an intrinsic calcium variable, which depends on the recent spiking activity. On a long time-scale, the weight values of the plastic synapses drift toward one of two possible states, depending if their value is above or below a weight-threshold parameter. The synapses therefore are bistable and robust to input activity and state-dependent variability. Equations (1) and (2) formalize the plasticity weight update mechanism that operates on short time-scales:

(1) $$\begin{array}{r} \begin{array}{c} w_{i}=w_{i}+\Delta w^{+},\text{if}\;V_{mem}\left(t_{pre}\right)>\theta_{mem}\;\text{and}\;\theta_{1}<\text{Ca}\left(t_{pre}\right)<\theta_{max}; \end{array} \end{array}$$

(2) $$\begin{array}{r} \begin{array}{c} w_{i}=w_{i}-\Delta w^{-},\text{if}V_{mem}\left(t_{pre}\right)<\theta_{mem}\;\text{and}\;\theta_{1}<\text{Ca}\left(t_{pre}\right)<\theta_{max}. \end{array} \end{array}$$

Here, *w*_*i*_ is the synaptic weight of a plastic synapse; the terms Δ*w*^+^ and Δ*w*^−^ determine the amplitude of the weight's increase and decrease, respectively. *V*_*mem*_(*t*_*pre*_) is the post-synaptic neuron's membrane potential at the time of the pre-synaptic spike arrival. If *V*_*mem*_ is above the threshold *θ*_*mem*_, the post-synaptic neuron must be about to spike, leading to the temporal sequence of pre- and post-synaptic spikes that leads to potentiation of the synapse (following to the “classical” spike-timing dependent plasticity rule), whereas if *V*_*mem*_ is below the threshold, the post-synaptic neuron is likely to have just spiked, leading to temporal sequence of spikes that correspond to depression of the synapse. The Ca variable represents the neuron's Calcium concentration, which is proportional to the neuron's recent spiking activity. This is the variable representing the “third factor” in this three-factor learning rule that gates plasticity (Neftci, 2018). The parameters *θ*_*min*_, *θ*_2_, and *θ*_*max*_ are the thresholds that determine in which conditions the weights are updated.

The long-term drift that determines the synaptic weight bistability properties, and which is superimposed to this STDP plasticity rule, is governed by the following equations:

(3) $$\begin{array}{r} \frac{d}{dt}w_{i}={\text{C}}_{\text{drift}},\text{if}\;w_{i}>\theta_{w}\;\text{and }w_{i}<w_{\text{max}}; \end{array}$$

(4) $$\begin{array}{r} \frac{d}{dt}w_{i}=-{\text{C}}_{\text{drift}},\text{if}w_{i}<\theta_{w}\;\text{and}\;w_{i}>w_{\text{min}}, \end{array}$$

where *C*_*d*_*rift* determines the rate of the drift, *θ*_*w*_ is the weight threshold that determines the direction of the drift, and *w*_min_, *w*_max_—the value of the high and low weights, respectively. Thus, plastic synapses are binary in the long term.

**Figure 2:**
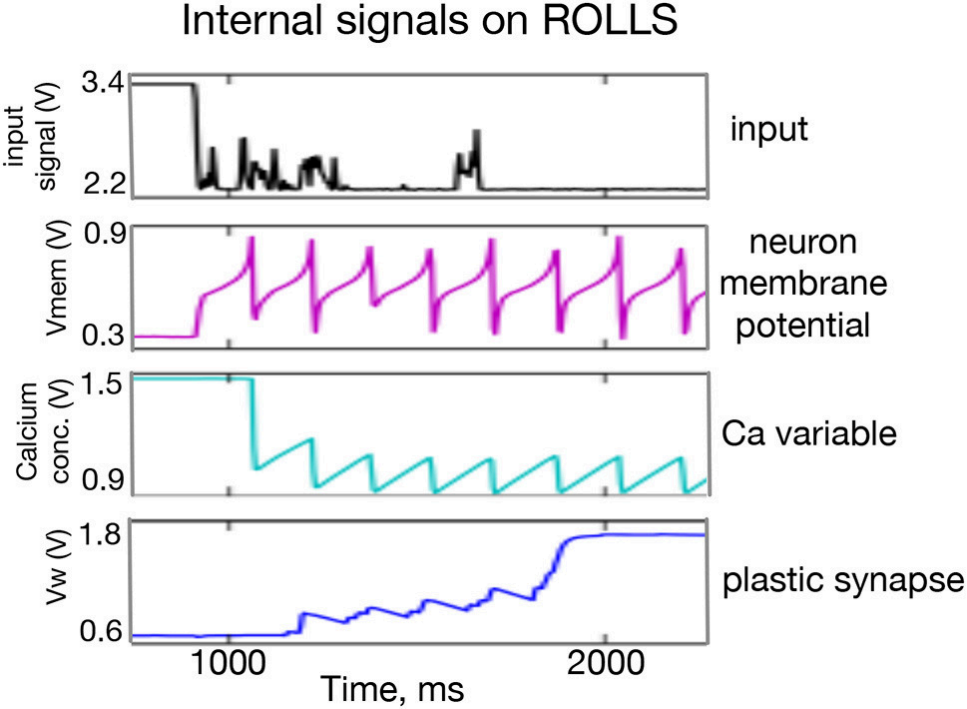
Oscilloscope traces of subthreshold activity of a silicon neuron and a plastic synapse. From **(Top to Bottom)** the input signal to the neuron with a number of (random) spikes, low-pass filtered by the “virtual” synapse; the neuron's membrane potential; a variable that models the neuron's calcium concentration; and the synaptic weight of a plastic synapse to which another external input is applied. One can observe potentiation of the plastic synapse after each spike of the monitored neuron and diffusion of the synapse's weight to a high value after it reaches a certain threshold.

Figure 2 shows traces of different components of an active neuron on the ROLLS chip and one of its plastic synapses, whose weight increases in real time in response to a constant input. A thorough description and characterization of the circuits can be found in Qiao et al. (2015).

Additional peripheral analog/digital input-output circuits for both receiving and transmitting spikes in real-time on- and off-chip follow an Address-Event Representation (AER) protocol (Boahen, 1999) and can be used to stimulate individual synapses on the chip. An on-chip programmable bias generator, optimized for subthreshold circuits allows the user to create networks with different properties and topologies and to program the properties of the synapses and neurons (such as time constants, leak currents, etc.).

The ROLLS was fabricated using a standard 180 nm CMOS 1P6M process. It occupies an area of 51.4 mm^2^ with approximately 12.2 million transistors.

### 2.2. Neuromorphic implementation of a soft-WTA network

Implementing a neural architecture, or model, on the ROLLS chip involves two steps. First, the non-plastic synapses are configured to create the static part of the neural network. For each synapse, one of four available weights can be selected, alongside with their type (inhibitory or excitatory). In the second step, the parameters of the neuron and synapse circuits are selected by setting the on-chip biases. These parameters are globally shared by all neurons and synapses.

**Figure 3:**
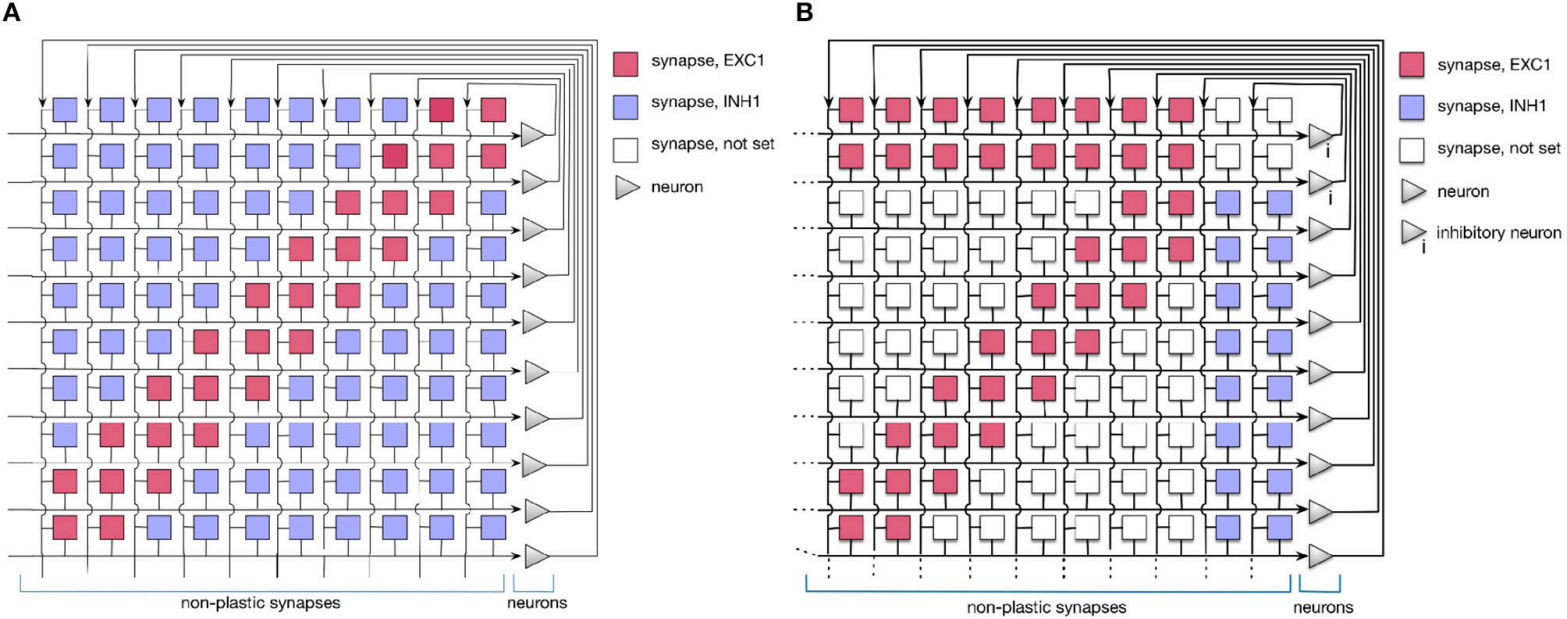
Setting the soft-WTA connectivity on the ROLLS chip. **(A)** Connectivity using excitatory synapses to the neuron itself and to its nearest neighbors and inhibitory synapses to all other neurons. **(B)** Connectivity scheme with excitatory projections of neurons to themselves, their nearest neighbors, and a small inhibitory group. The latter inhibits the whole excitatory population.

In this work, we use a simple 1D Dynamic Neural Field (DNF) (Schöner and Spencer, 2015) to represent items in a sequence. On the neuromorphic chip, such DNF can be realized using WTA connectivity pattern (Sandamirskaya, 2014). Such WTAs are considered building blocks for cognitive neuromorphic architectures (Neftci et al., 2013). In fact, two connectivity patterns can be used to realize a WTA network on the ROLLS chip, as shown in Figure 3. The red and blue squares in the figure represent excitatory and inhibitory synapses that connect neurons (triangles on the right). Tables A1 and A2 present a list of exemplary biases for neural and synaptic circuits, respectively, that generate winner-take-all behavior in a configuration presented in the Figure 2B.

In order to realize a soft-WTA behavior (the term *soft* indicates that a group and not only a single neuron wins the competition) on the ROLLS chip, two types of connectivity networks can be configured.

In both settings, local groups of neurons can stabilize their activity by excitatorily projecting to themselves and to their nearest neighbors. Global inhibition ensures that only one group is active at a time and keeps the network activity from spreading across the whole population. Stable activity bumps can be achieved with two connectivity patterns in a population of spiking neurons. In the first pattern (A), we define the extent of region around each neuron, in which it will be connected to its neighbors with excitatory synapses. Every connection exceeding this excitatory range will form an inhibitory synapse (see Figure 3A). In the second pattern (B), a separate group of inhibitory neurons is introduced, which task is to suppress the activity of the excitatory population (Figure 3B). In this case, all excitatory neurons are positively coupled to the inhibitory group, which inhibits them back.

Evidence from neuroscience suggests that inhibition is provided by a separate set of inhibitory interneurons in many cortical areas (Couey et al., 2013; Buetfering et al., 2014). Thus, a WTA network with a separate group presented in Figure 3B appears to be more biologically plausible. However, from an engineering point of view, the choice may depend on other considerations, e.g., if the number of available synapses per neuron is limited, pattern (B) maybe preferable, while if rather the number of neurons is the bottleneck, the pattern (A) might be more advantageous. One should also consider that the additional inhibitory group adds a delay to the inhibitory feedback and may render parameter tuning more challenging.

### 2.3. Neuromorphic architecture for sequence learning

In this work, an architecture for serial order memory proposed in the framework of dynamic neural fields (Sandamirskaya and Schöner, 2010b; Duran and Sandamirskaya, 2012), was realized in neuromorphic hardware in the following way.

**Figure 4:**
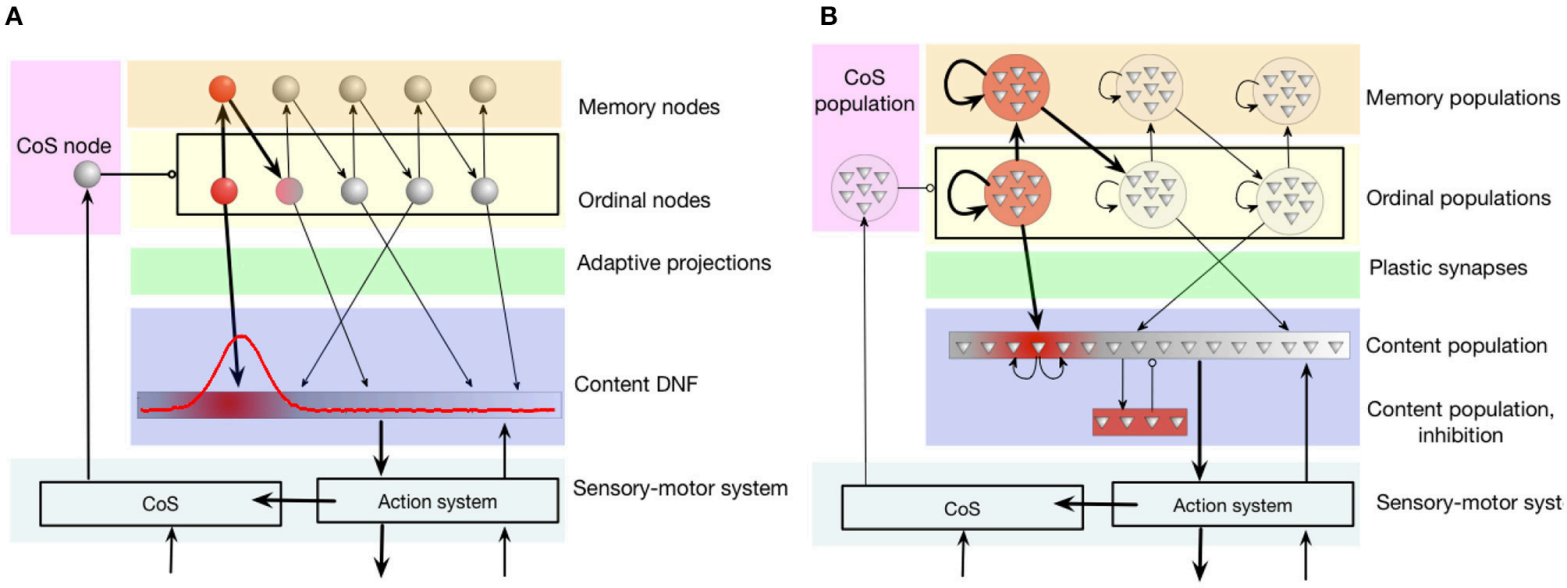
The serial order architecture, introduced by Sandamirskaya and Schöner (2010a). **(A)** The continuous version of the serial order architecture. A set of discrete neural-dynamic nodes represents ordinal positions within a sequence. The content Dynamic Neural Field (DNF) represents the perceptual or motor features of the stored items. A sequence of items is learned in adaptive connections between the ordinal nodes and the content DNF. **(B)** The neuromorphic realization of the architecture using populations of neurons. Note that in order to create stabilized peaks of activation that correspond to self-sustained activation of a neural population, neurons within a group need to be recurrently connected. CoS is the condition of satisfaction system that detects sequential transitions both during sequence learning and acting-out.

The connectivity pattern, shown in Figure 4A, includes a population of *ordinal nodes* (yellow region in the figure). Each ordinal node, if active, represents a position in a sequence. Ordinal nodes activate each other sequentially. This is achieved through a set of *memory nodes* (orange region in the figure): each ordinal node activates the respective memory node, which in its turn activates the *next* ordinal node. Memory nodes feature strong recurrent connections that lead to their self-sustained activity: memory nodes stay active even when the respective ordinal node is not active any more. Thus, the memory nodes “keep track” of the position in the sequence during sequential transitions (when ordinal nodes are inhibited, as will be shown below).

The *content dynamic neural field* (content DNF; blue region in the figure) represents the perceptual states and/or actions that can be associated with a sequential position (an ordinal node) with plastic synaptic connections (green region). The content DNF is connected to the action system of the agent and sets attractors to generate behavior. It also receives perceptual input during sequence learning that creates activity in this field that corresponds to the recognized, e.g., demonstrated, actions.

The *condition of satisfaction* (CoS) system (lilac region in the figure) detects when each initiated action or perceptual state has reached the intended outcome (Richter et al., 2012). To achieve this, the CoS node is driven by a perceptual module that receives input signaling the currently perceived or performed action and input from sensors, configured to activate the CoS node if the end of an action has been detected (e.g., end of presentation of the object during learning, or end of a goal-directed movement during replay).

When the CoS node is activated, it inhibits all ordinal nodes, in particular removing activation of the currently active ordinal node and thus stopping the learning process that was strengthening synaptic connection between this ordinal node and an active region in the content DNF. The activity in the content DNF will also cease in the transition.

During learning, this happens because transition means switching to the next item, which happens through sequence of “forgetting” and “detection” instabilities (the old object disappears and the new object appears), which leads to decrease of activation in the DNF. If an item was successfully learned and there's no perceptual input (Laser off), the ordinal population “recalls” the learned item in the activity in the content DNF. Since the DNF is connected to the CoS, successful learning (and not perceiving visual input) triggers the transition. When the laser is switched on the DVS_on population, driven by the visual input, becomes active and inhibits the CoS, next item can be learned.

During sequence replay, on the other hand, the activity peak in the content DNF is supported by the active ordinal node. When the CoS becomes active and inhibits the ordinal nodes, the activity in the content DNF also ceases. In both cases, the decrease of activity in the content DNF leads to deactivation of the CoS node, which releases the inhibition on the ordinal nodes. The next ordinal node can become active now, driven by the asymmetric connection from the previous memory node.

Figure 4B shows how this neural dynamic architecture can be realized with populations of spiking neurons—a step required for the implementation in neuromorphic hardware (Sandamirskaya, 2014). Several constraints have to be taken into account: (1) the limited amount of silicon neurons, (2) robustness to mismatch, and (3) shared parameter settings across all neurons that need to exhibit different firing behaviors.

To cope with mismatch, we used populations of 10-20 neurons to represent a neuronal node (ordinal, memory, or CoS nodes).

**Ordinal groups**: Each ordinal group contains 20 silicon neurons in our experiments, inter-connected via excitatory synapses in an all-to-all fashion. Silicon neurons in different ordinal groups inhibit each other, forming a WTA network. This allows only one ordinal group to be active at a time.

**Memory groups**: each ordinal group excites a corresponding memory group that contains 10 neurons. Memory groups remain active for the whole trial due to their high self-excitation. Each memory group has excitatory synapses to the next ordinal group. At the same time, they signal whether their corresponding ordinal node has already been activated, by slightly inhibiting it. This ensures that the ordinal node that has not yet been activated receives the highest excitatory input in the transition phase.

**Content group**: every content neuron is connected via plastic synapses with ordinal neurons with the possibility to strengthen synaptic weights toward an active region in the content WTA. Initially, all plastic synapses are depressed (have a low weight) and can only become potentiated once the ordinal and content neurons are co-active.

**CoS group** contains 10 neurons that are externally stimulated upon a keystroke in most experiments here in order to trigger a transition.

**Figure 5:**
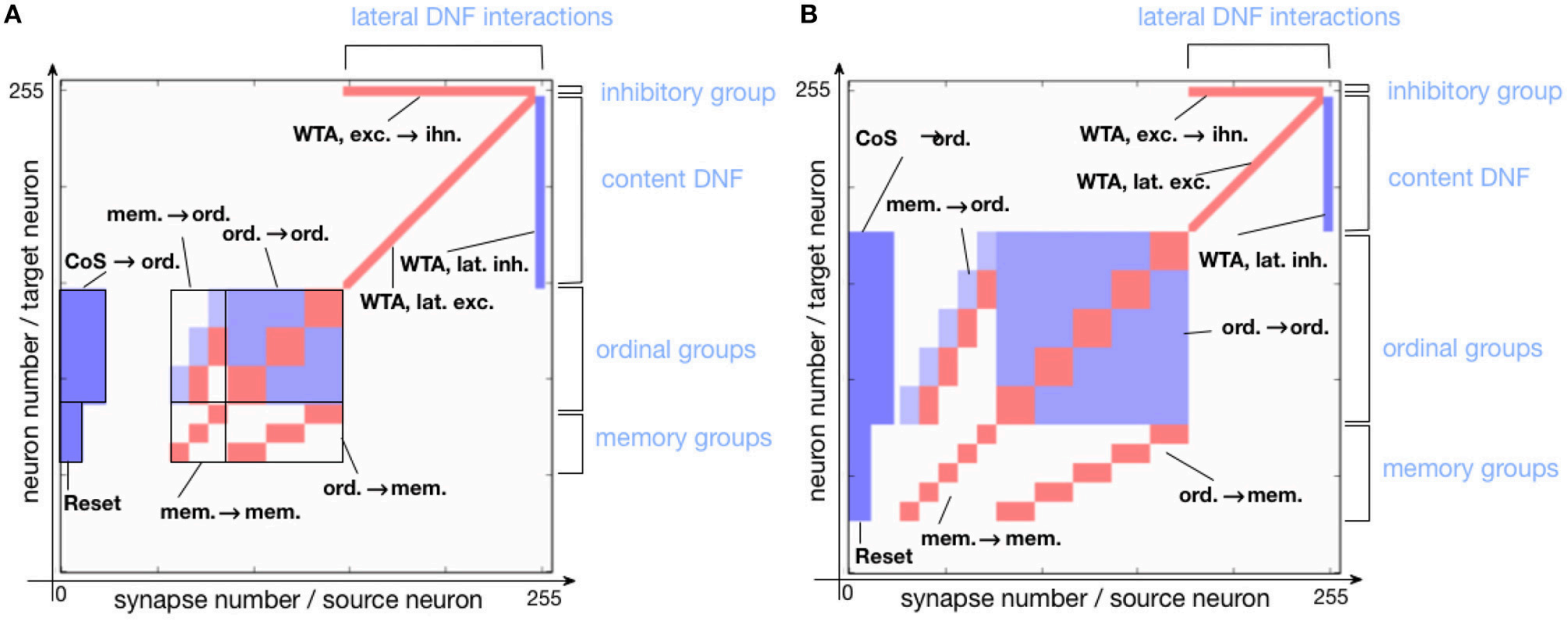
Connectivity matrix of non-plastic synapses, sent to the neuromorphic chip to encode the serial order architecture. Different shades of blue indicate different inhibitory synaptic weights (the darkest being the strongest). Red represents excitatory synapses, which all have the same weight. **(A)** Connectivity matrix for storing a sequence of three items. **(B)** Connectivity matrix for storing a sequence of five items.

Figure 5 shows the connectivity matrix of non-plastic synapses, set on the neuromorphic hardware to realize the sequence learning architecture. Figure 5A shows the implemented architecture for storing a sequence of three items and Figure 5B shows the connectivity matrix for storing a sequence with five items. Note that the hardware used here is a research prototype with only 256 analog neurons, which, along with mismatch, limits the number of items that the system can store. For this reason, the size of the content DNF is reduced for the 5-items architecture.

**Figure 6:**
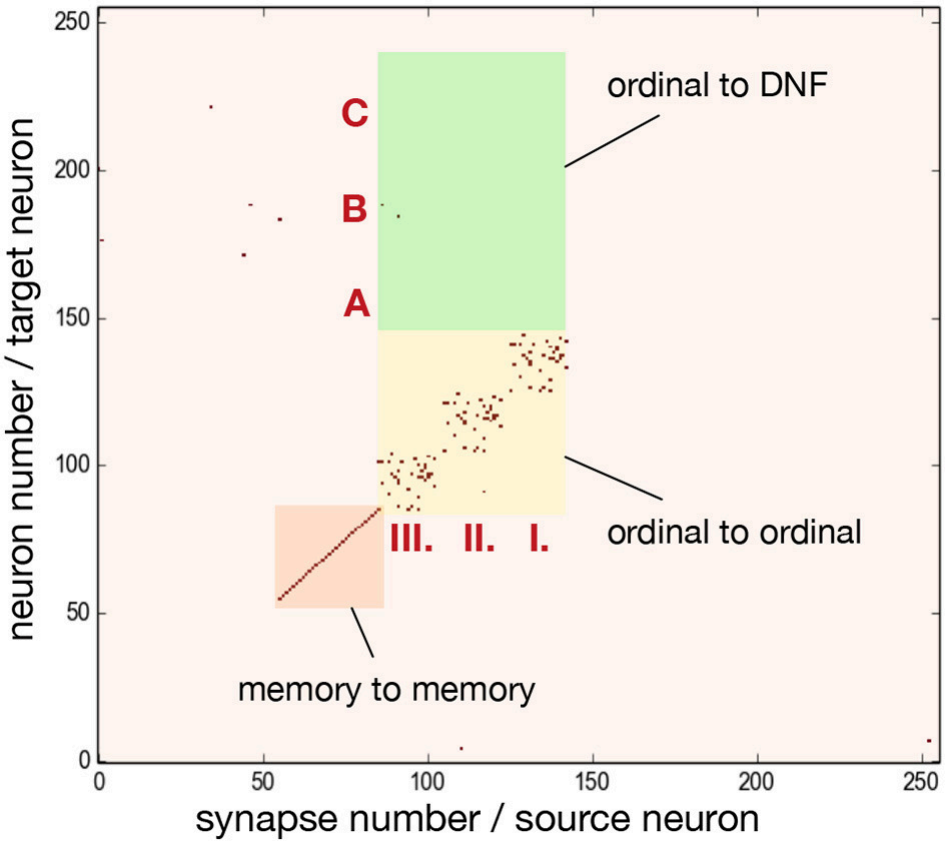
Initial state of the plastic synapses for a sequence of three items before sequence learning, as measured from the ROLLS chip. I., II., III. mark pre-synaptic neurons that belong to the ordinal groups; A, B, C mark post-synaptic neurons that belong to different regions of the content DNF. Red dots show potentiated synapses, used for additional strengthening of recurrent connections in the ordinal and memory groups. These synapses don't participate in learning a sequence. Synapses in the green region connect ordinal nodes to content DNF and will represent the sequence after learning. These plastic synapses are depressed (low) at initialization. The plastic synapses are read-out by activating them sequentially and observing activity of the post-synaptic neuron.

To achieve the desired behavior of the architecture, parameters specifying neuron and synapse dynamics have to be set in order to meet certain requirements posed on neural populations. Since ROLLS chip features only 4 different excitatory weights, we additionally used potentiated plastic synapses to strengethen connections within ordinal and memory groups. Figure 6 shows the plastic synapse matrix after initializing a network for storing a sequence of three items. Each red dot shows a potentiated (high) plastic synapse at initialization of the architecture (before learning). The diagonal of potentiated synpases for neurons 62–92 (orange region) and 93–153 (yellow region) in Figure 6 corresponds to the additional self-excitation in the memory groups and ordinal groups, respectively, using potentiated plastic synapses. Non-plastic connectivity in these groups is enhanced by using 30% of randomly potentiated plastic weights. Since state of the plastic synapses can not be read-out directly, we used a protocol to read them out, in which each synapse is stimulated and activity of the post-synaptic neuron is read-out, at turned-off plasticity.

## 3. Results

### 3.1. Storing a three-items sequence

**Figure 7:**
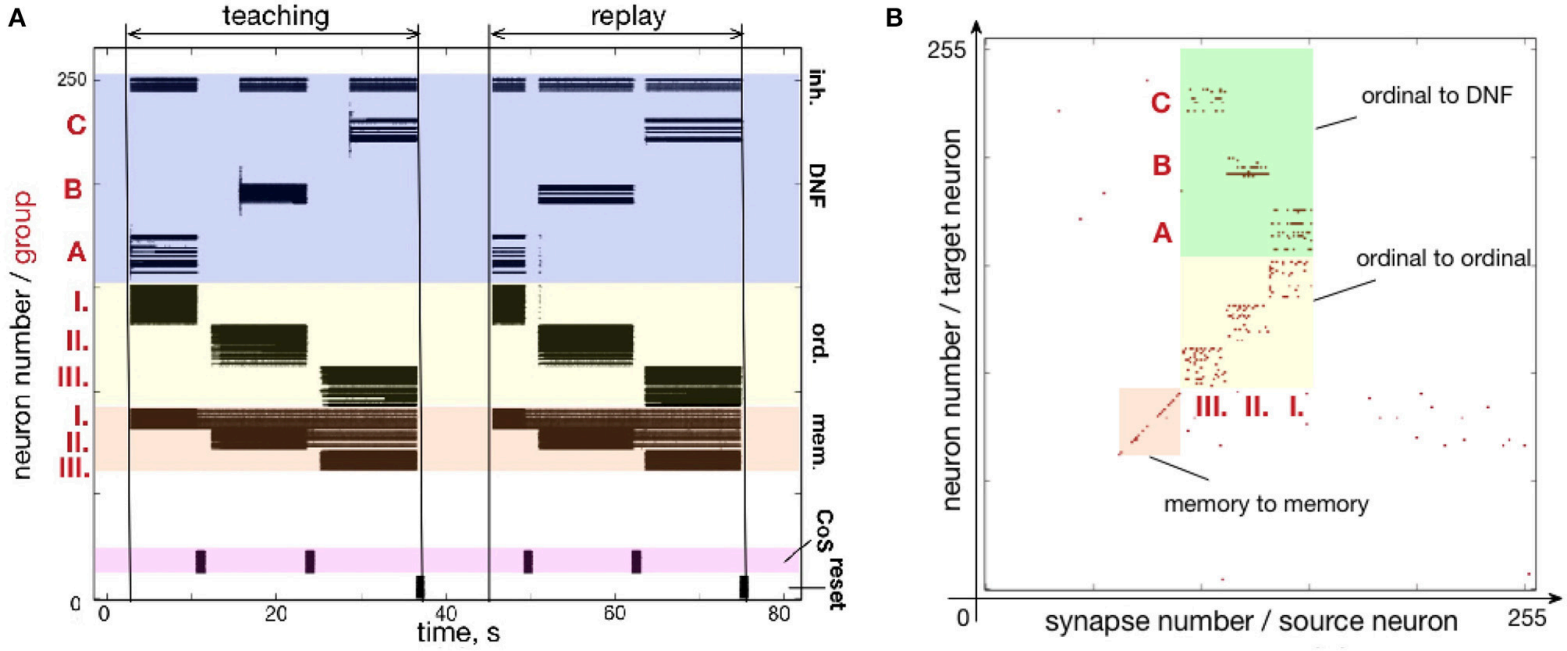
Learning a three-items sequence. **(A)** Time-course of neuronal spiking activity (raster plot) for the sequence A-B-C. Numbers I., II., III. mark neurons of memory and ordinal populations; letters A, B, C mark three regions on the content DNF; CoS – condition of satisfaction neurons that trigger sequential transitions. In the teaching period, the sequence A, B, C is activated in the content DNF by an external input. In the replay period, this sequence is reproduced by activity flowing from the ordinal nodes to the content DNF through the potentiated plastic synapses. Note that memory nodes stay active until the end of the sequence, when they are inhibited by the Reset population, both during teaching and replay. Active memory nodes represent progress along the sequence. **(B)** Weights of the plastic synapses on the ROLLS chip after learning the sequence A-B-C. Potentiated synapses in the green region show learned associations from the ordinal groups to the content DNF: I.-A, II.-B, and III.-C.

Figure 7 shows how a simple sequence of three items can be successfully learned and recalled on neuromorphic hardware. Here, the architecture is presented with a sequence of items A-B-C and stores them in plastic synapses that connect ordinal groups (I., II., III.) to the content DNF.

Figure 7A shows the raster plot of spikes recorded from the neuromorphic chip ROLLS. Each black dot corresponds to a spike, emitted by one of the 256 neurons on the ROLLS chip. Vertical axis shows neuron index (0–255), horizontal axis shows time in seconds. Colors mark different populations of neurons on the chip, according to the serial order architecture (Figure 4): from bottom to top, lilac is the CoS population, orange are the memory groups, yellow are the ordinal groups, dark blue is the content WTA / DNF (its excitatory and inhibitory parts).

The neural activity is initially induced by an external input according to the following stimulation protocol. First, the sequence gets “launched" by the stimulation of the first ordinal group of neurons for 3,000 ms with 200 Hz spiking input applied via virtual synapses (the “go" signal). The transition signal between sequential items, which suppresses the activity of ordinal neurons, is triggered by stimulating the CoS group for 500 ms with 800 Hz. An input to the content neurons, which will be replaced with sensory input in section 3.1, consists of Poisson spike trains with firing rate distributed along the content population according to a Gaussian-function, centered at a selected “current item” neuron (marked with A, B, or C in Figure 7A), with the maximum of 900 Hz and a standard deviation of 5 neurons. All content neurons additionally receive random inputs between 0 and 10 Hz to simulate sensory noise. During learning, the content neurons are stimulated for 6,000 ms for each item in the sequence.

The WTA connectivity in the content population leads to formation of a localized “activity bump”: noise is suppressed by global inhibition and activity within the Gaussian is stabilized by the recurrent excitation. The neural location of the activation peak defines the content (A, B, or C here) and leads to strengthening of the connections between the active region in the content population and active neurons in one of the ordinal populations, according to the on-chip learning rule.

After learning the full sequence, an external input is sent to the inhibitory *reset group* which suppresses activity of the memory groups, resetting the ordinal system of the architecture. After the complete suppression of the neural activity in the ordinal system, the recall is triggered by an external stimulation of the first ordinal group (the “go” signal). Transitions between states are initiated by an external stimulation of the CoS group and can take place at arbitrary moments in time (as can be seen in the “replay” phase on the raster plot of Figure 7A). This transitions can be triggered by sensory input that signals the end of an action, associated with items in the sequence (where action can also be intrinsic, like an attention shift), or can be generated internally, e.g., by a neuronal “timer,” as was introduced by Duran and Sandamirskaya (2018). The serial order architecture leads to a sequential activation of the ordinal groups, which, in their turn, lead to sequential activation of the stored locations on the content population, through the on-chip plastic synapses.

Note that memory groups keep firing until the end of the teaching or replay period, keeping track of the unfolding sequence. This activity is achieved by strong recurrent connections in the memory groups and can be used to monitor sequence learning and replay by a higher-level system in a hierarchical sequence representation architecture (Duran and Sandamirskaya, 2012).

Figure 7B shows plastic synapses on the ROLLS chip after learning. Here, each red dot corresponds to a potentiated plastic synapse. As mentioned previously, the portion of plastic synapses within the yellow and orange colored regions were set to be potentiated in order to increase self-excitation in the ordinal and memory neural populations and did not participate in learning. Plastic synapses in the green region are the ones connecting the ordinal groups to the content WTA and these synapses are depressed (set to zero) initially and are potentiated during sequence learning. Note that there is no direct access to the state of the plastic synapses on the ROLLS chip, thus to create Figure 7B, the potentiated synapses were read out by stimulating each plastic synapse and measuring if the stimulation lead to a postsynaptic spike.

One can observe the potentiated synapses between ordinal group I and item A, ordinal group II and item B, and ordinal group III and item C. One can also notice a considerable amount of noise in the plastic synapses (within green region, but also across the chip). Despite this noise, the system is capable to reproduce the A-B-C sequence, thanks to the WTA dynamics of the content population (seen in the “replay” part on the raster plot Figure 7A).

### 3.2. Storing a five-items sequence

**Figure 8:**
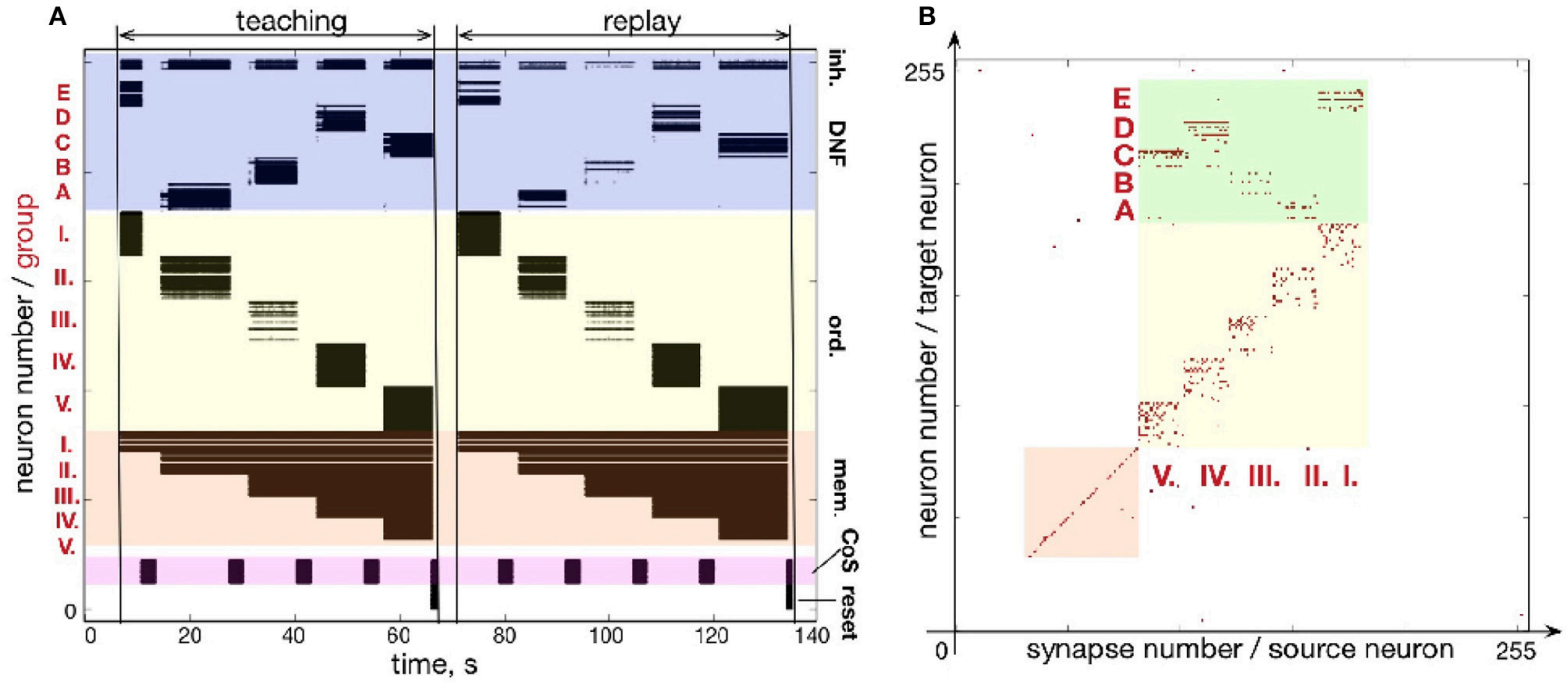
Learning a five-items sequence. **(A)** Raster plot of the spiking activity on the ROLLS, when the sequence E-A-B-D-C is learned (“teaching”) and reproduced (“replay”). Colors mark different neuronal populations (see also Figure 7). **(B)** Weights of plastic synapses after learning the sequence. Plastic synapses in the green region encode the sequence: I.-E; II.-A; III.-B; IV.-D; V.-C.

Similar to Figures 7, 8 shows how a sequence of five items can be learned and reproduced on the ROLLS chip. Here, the sequence E-A-B-D-C is stored in the plastic synapses on the ROLLS chip during the “teaching” period (Figure 8A) and is reproduced by the chip without external stimulation in the “replay” period. As for the three-items sequence, the external inputs were used to start both the teaching and the replay periods and to trigger transitions between sequential elements at arbitrary moments in time (both during learning and replay). During learning, inputs to the content WTA that correspond to different items (E-A-B-D-C) were introduced externally (as spike trains with firing rate profile shaped according to a Gaussian, centered over selected location on the content DNF). During replay, activity in the content DNF is induced through the potentiated plastic synapses from the ordinal nodes to the content DNF.

Figure 8B shows the plastic weights on the ROLLS chip after learning. Again, potentiated synapses (red dots) in the yellow and orange regions are just auxiliary weights we used to support recurrent connections within ordinal and memory groups. Learned synapse are red dots in the green region and correspond to synapses between ordinal groups (I.-V.) and different regions (A-E) on the content DNF.

Although the learned representation of the sequence is noisy, the system is capable of correctly recalling the stored sequence. The ordinal nodes activate the locations on the WTA population in the memorized order, with transitions triggered by the (externally stimulated) CoS population. In the recall session, the content WTA/DNF does not receive any external stimulation. Activity of neurons in the content DNF is solely triggered by the formed (learned) associations (potentiated plastic synapses) connecting subsets of ordinal and content populations.

**Figure 9:**
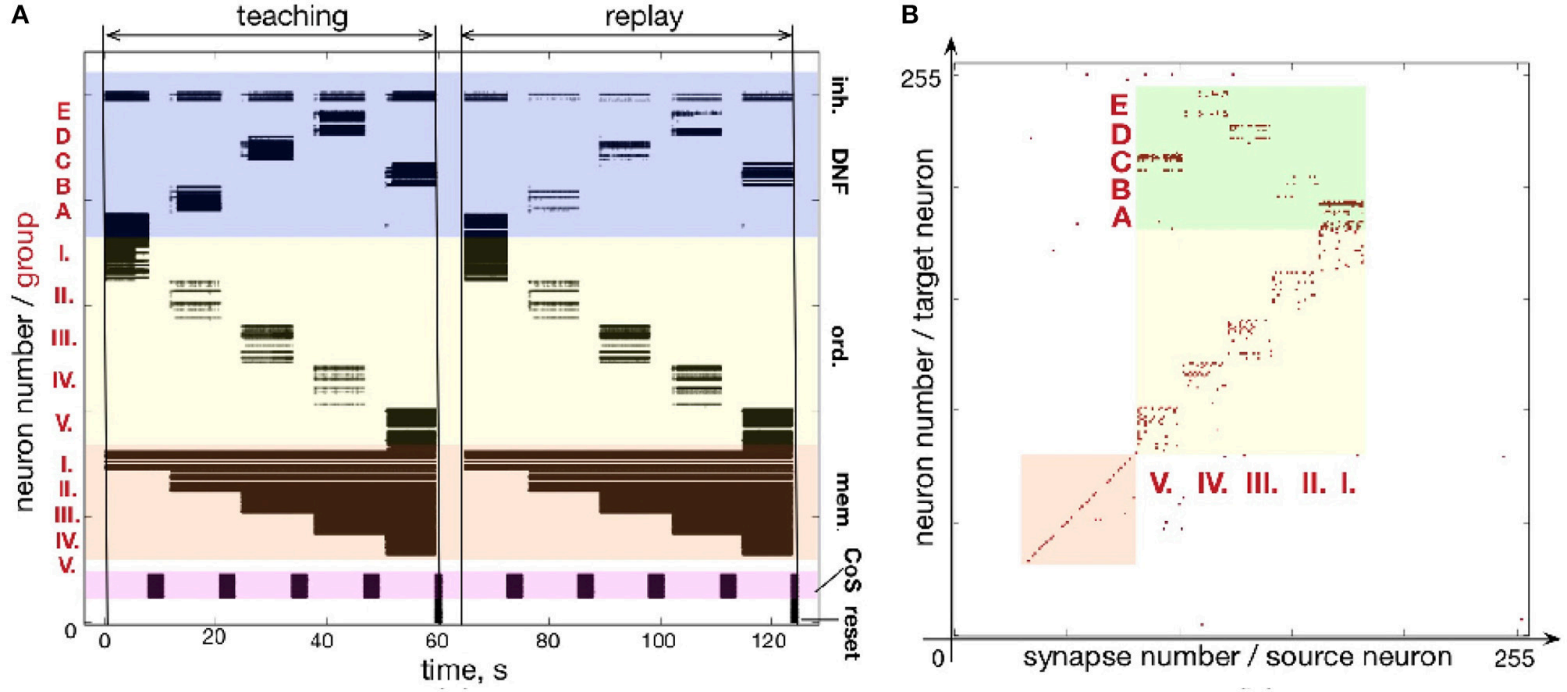
Learning sequence A-B-D-E-C. **(A)** Time-course of spiking neuronal during learning and recall (similar as Figure fig:EABDC). **(B)** Plastic weights after learning the sequence. Weights in the green region encode the sequence by storing the associations between neuronal groups: I.-A; II.-B; III.-D; IV.-E; V.-C.

Figure 9 shows another example of a five-items sequence (A-B-D-E-C) learned on the chip. By using smaller population sizes, longer sequences can potentially be learned. However, mismatch will become more noticeable by making the network prone to commit serial order errors.

### 3.3. Repeating items

**Figure 10:**
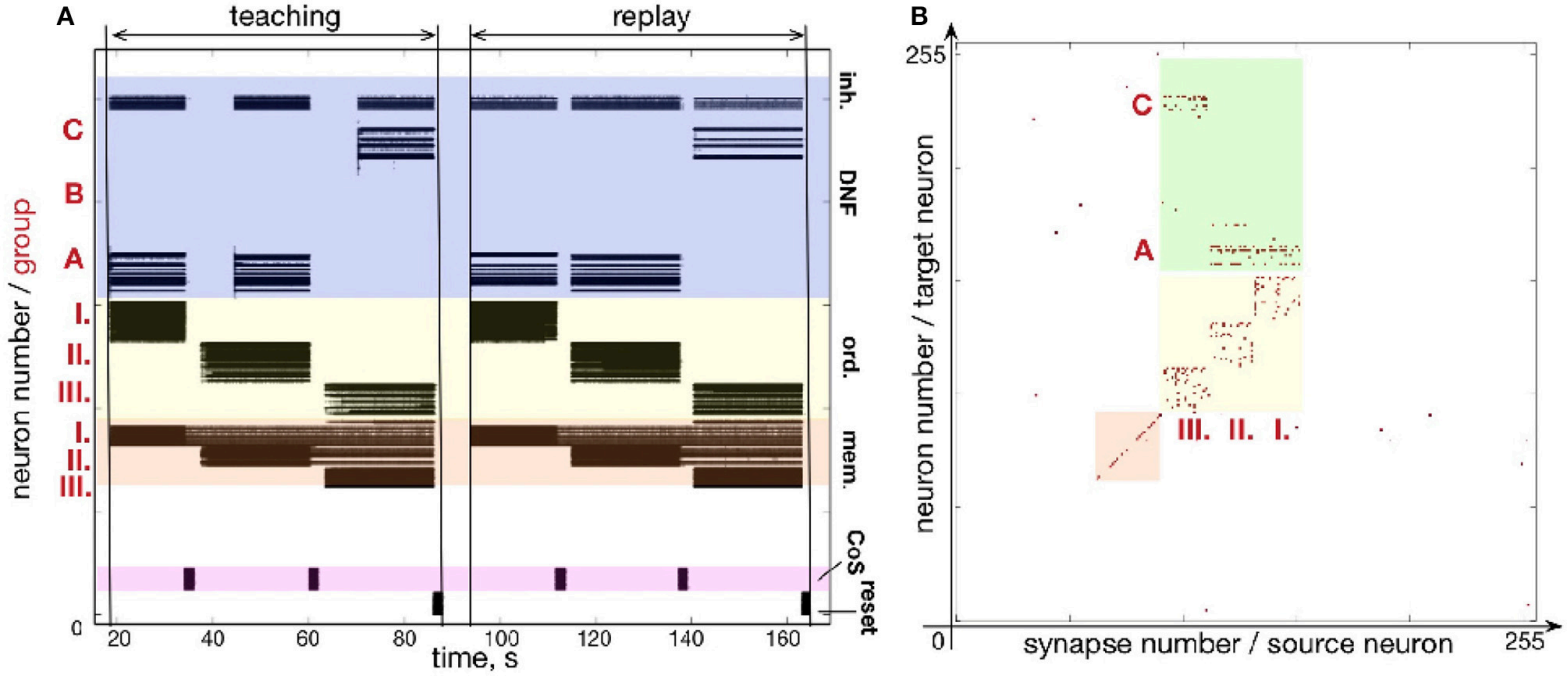
Learning a sequences with repeated items: **(A)** Time-course of spiking neuronal activity for learning and reproducing the sequence A-A-C. **(B)** Plastic weights after learning the sequence AAC, storing the sequence: I.-A; II.-B; III.-C.

Figure 10 shows that sequences with repeating items can be stored on the chip. Here, a sequence A-A-C is learned and reproduced on the chip. Often, the serial order architectures, in which sequence is represented by direct connections between items' representations, have difficulties with sequences with repeated items. In our simple 3-items example, for instance, item A would have to be connected both to itself and to the item C in order to represent the A-A-C sequence. Additional mechanism would be needed to distinguish between the first and the second occurrence of A. Our serial order architecture features a “positional” (i.e., spatial) representation of serial order (Sandamirskaya and Schöner, 2010a) and does not suffer from this problem.

As can be seen in Figure 10B, the plastic weights that connect ordinal group I to element A and ordinal group II to element A are independent of each other and sequences with arbitrary number of repeating elements in any position in the sequence can be stored in this way.

Additional handling for repeated items is often required when sequences are learned in recurrent neural networks, e.g., Hochreiter and Schmidhuber (1997). In our architecture, to the contrary to these networks, all input representations are stored in the form of independent associations formed between the DNF content and the ordinal nodes. This feature distinguishes this implementation from other neural architectures designed to store sequences.

### 3.4. Unlearning a sequence

Another property of the presented serial order architecture is its ability to forget and update previously learned sequences. When a sequence has been learned, activation of an ordinal node induces activation of a certain region on the content DNF, through the plastic synapses. A strong external input to the content DNF, however, can overcome this activation: the winner-take-all connectivity of the content population induces competition between the old item and a new item. If the external input is strong enough to overcome the global inhibition of the content DNF, new activity peak will be created. This new activity peak, in its turn, suppresses the activity induced through the learned plastic synapses. Thus, the synapses that conduct external input to the content DNF need to be stronger than the potentiated plastic synapses for such reset to work (input conducted through plastic synapses can not overcome global inhibition from the strongly supported activity peak, induced by the external input).

When a new activity peak is formed in the content DNF, the learning rule leads to potentiation of the plastic synapses leading from the active ordinal node to the newly activated region on the content DNF. At the same time, the synapses to the old item will be slowly “forgotten,” because of the synaptic depression working on synapses with post-synaptic neurons having low (here, zero) firing rate. Thus, the new sequence can be learned, while the old sequence will slowly be forgotten using the synaptic depression of the on-chip learning rule (Brette et al., 2007).

Forgetting becomes possible because of the specific learning rule implemented on the ROLLS device: The weight of the plastic synapse is updated upon the arrival of a pre-synaptic spike. Forgetting takes place whenever a pre-synaptic spike is not followed by a post-synaptic spike. In this case, the weight of the plastic synapse gradually decreases and eventually reaches a low (depressed) state.

**Figure 11:**
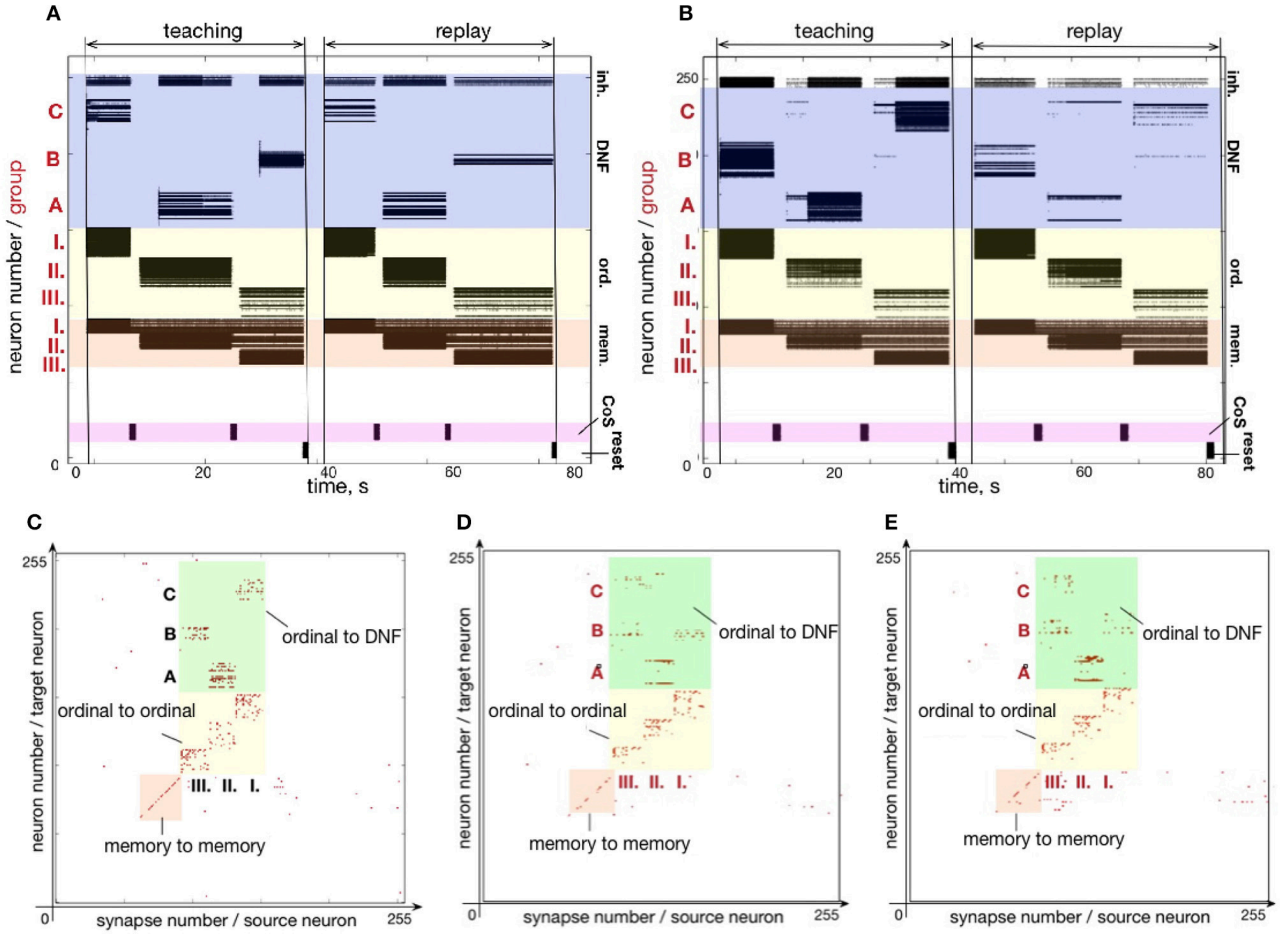
An experiment to demonstrate unlearning: **(A)** spiking activity on ROLLS during learning and replay of a sequence C-A-B; **(B)** learning and replay of a new sequence, B-A-C, without resetting the plastic weights; **(C)** the plastic weights after learning the first sequence (C-A-B); **(D)** plastic weights after the first trial of learning the second sequence, B-A-C; **(E)** plastic weights after the fourth trial of learning the second sequence.

An experiment designed to test the forgetting mechanism is shown in Figure 11. Figure 11A shows the firing activity when sequence C - A - B is memorized and recalled. Figure 11C shows the state of the plastic synapses after learning. After recalling the sequence C - A - B, we stimulated the content DNFs with items in a different order: B - A - C. The firing activity during learning and recalling the new sequence B - A - C is shown in Figure 11B. The resulting synaptic weight matrix is shown in Figure 11D.

In this figure we can see that the plastic synapses that formed between the first ordinal group and the item C get depressed after stimulating the content neurons with the new sequence B - A - C a single time (compare with Figure 11C). Instead, the first ordinal group strengthens synapses toward the recently stimulated content neurons B. However, traces of potentiated synapses between content B and ordinal group III remain potentiated after the single trial of learning the new sequence B - A - C. Synapses toward the correct element C in the position III become potentiated gradually.

The weight matrices in Figure 11D show that the previously learned element B remains more consolidated than the newly learned element C. However, Figure 11B shows that the high activity of the externally stimulated content neurons eventually (after 4 trials) completely suppresses the activity of the previously learned sequence. Hence, during recall the neurons' firing activity resembles only slightly the old sequence (meant to get forgotten and overwritten) and more strongly represents the new sequence.

Figure 11E shows plastic weights after stimulating the content layer with the items in order B - A - C four times. The new sequence B - A - C is successfully learned and the old sequence C - A - B is almost completely “forgotten.” A small trace of the previous sequence remains, which is suppressed by the WTA dynamics of the content DNF during replay.

### 3.5. Robotic implementation: learning a sequence of visual stimuli

In this section, we present an implementation of the serial order architecture on the neuromorphic chip interfaced to a robotic platform. Here, the ROLLS processor is configured with a similar connectivity as for the experiments with artificial external inputs. The only difference is that we found a configuration, in which the 30% of randomly potentiated plastic weights used in the ordinal and memory populations to enhance non-plastic self-excitation was not required (bias parameters, used here are listed in Table A2, Exp.2). The content DNF population here receives input from a neuromorphic camera Dynamic Vision Sensor (DVS) (Lichtsteiner et al., 2006; Liu and Delbruck, 2010), mounted on top of a robotic vehicle (Conradt et al., 2009)^1^.

Each pixel of the eDVS is sensitive to the temporal change in luminance and signals events when such change exceeds a threshold. The change events are communicated off-sensor using the Address Event Representation (AER) protocol, typically used for spike-based communication.

The DVS sends events directly as input spikes to the virtual synapses of the content neurons. Each event is a tuple of pixel coordinates (x,y) and polarity (pol), which signals whether the detected brightness change is positive or negative (we don't distinguish between events with different polarities here). Each DVS event triggers stimulation of a neuron in the content DNF according to the x coordinate of the active pixel^2^. As a sensory stimulus, we used a blinking laser pointer, highlighting different points of the scene, perceived by the robot. The reflection of the laser pointer generates a large number of DVS events that allow the content WTA to easily filter out sensory noise and form activity bump over the highlighted region. This allows us to avoid any sophisticated visual preprocessing.

Observing the highlighted region at different positions in the DVS's field of view creates activity at different locations of the content WTA on ROLLS. Simultaneous high activity in the content DNF population and an active ordinal population lead to potentiation of plastic synapses between these populations during sequence learning. During sequence recall, activity of the ordinal populations is transmitted to the content DNF population over the potentiated synapses for each sequential item. The active regions of the content WTA are read-out to set the turning angles for the robot (we hard-coded this mapping here for simplicity, although DNF-based navigation principles could be used to drive robot's movement more directly, as we have demonstrated previously, Blum et al., 2017; Milde et al., 2017a,b). Active content WTA neurons initiate turning to the stored directions in the order of the learned sequence.

**Figure 12:**
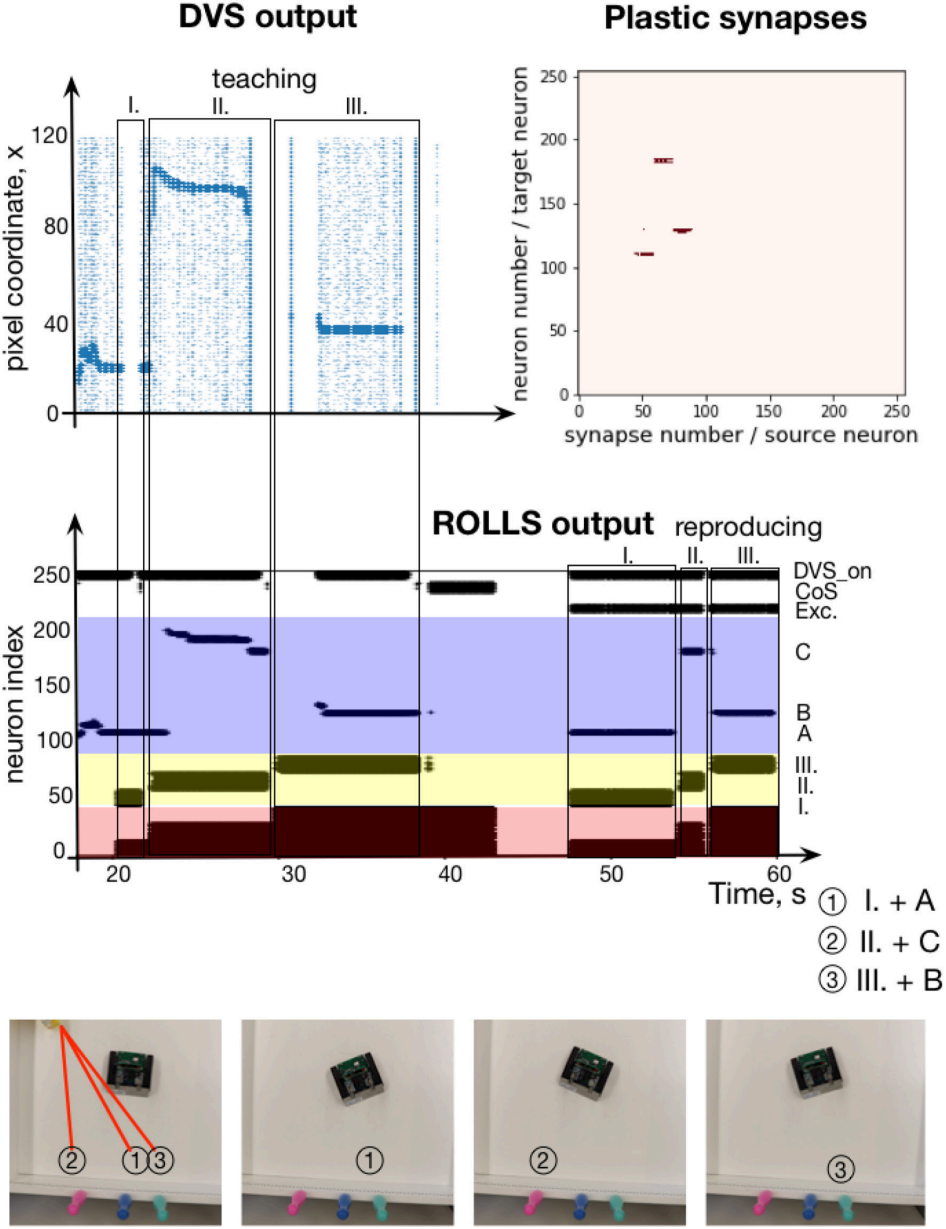
Learning a sequence of cued locations with a robot. **(Top, left)** The output of the Dynamic vision Sensor (DVS) camera of the robot: events from rows of the DVS over time. Regions with high activity correspond to horizontal positions of locations, cued with a laser pointer. **(Top, right)** Plastic synapses after learning. Dark red dots are synapses with high weights (only synapses from ordinal populations to the content DNF are probed here). **(Middle)** Spiking activity of neurons on the ROLLS chip during the robotic sequence learning experiment, in which sequence of three locations was learned (A-C-B) and reproduced by turning to center respective location in the field of view of the robot's DVS (the mapping from position in the camera's FoV and angle of rotation was hard-coded here for simplicity). **(Bottom)** Snapshots of the experiment from an overhead camera. See main text for details.

The experimental setup is shown in Figure 12, bottom. The pushbot learns the sequence of angular locations of the highlighted regions. The middle plot on the Figure 12 shows the resulting spiking activity of this experiment. During learning, the content WTA neurons are activated by DVS events shown in Figure 12, top, left. This plot shows the DVS events from each column of the sensor over time during the learning phase. The most salient input is amplified (neurons activated by the laser pointer reflection), whereas noise is suppressed due to the global inhibition in the DNF / WTA network.

In this experimental example, sequence transition is initiated by activation of the CoS group when the laser pointer is switched off before it is moved to the new position. This creates a clear sequential structure of the task. With a more sophisticated vision processing available, transitions could be triggered by the lack of an overlap of the highlighted region and segmented object when the laser pointer is moved from one object to another one without switching it off.

Whenever the laser pointer is switched off, the CoS population is activated. We achieved this by introducing a neural population that is activated by the strong input from the DVS (irrespective of its position), DVS_on population in Figure 12. This population inhibits the CoS population, which is otherwise activated by the output of the content DNF. When the laser pointer is switched off, the DVS_on population yields its activation and the CoS is activated briefly. The CoS population suppresses all ordinal nodes until the laser is turned on again, activating the DVS_on population. The next ordinal group is then activated, driven by the connectivity of the serial order architecture. The ordinal populations are consequently activated in a sequence and strengthen their synapses to different active content neurons.

The learning phase is followed by a global reset that suppresses the activity of the memory nodes, which otherwise keep track of the unfolding sequence. A sequence recall is triggered by a “go” signal—external activation of the first ordinal neural population and a new population, which sustains its activity through the whole experiments and excites the DVS_on population in order to suppress the CoS signal. The target positions, stored in plastic synapses during learning, are reproduced and drive the robot to turn toward the orientation that puts the cued positions in the center of field of view of the DVS. Once the movement execution toward the recalled orientation is finished, the DVS_on population is inhibited externally leading to activation of the CoS signal and thus a transition in the sequence.

Figure 12, top-right shows the read out of the plastic weights after learning the visual sequence. Here, to visualize the strengths of the plastic connections, the ordinal neurons are stimulated one by one and activity of the whole chip is observed. In particular, we can see that three different positions in the content populations get activated when associated ordinal groups are stimulated. This measurement is based on whether or not a spike of a particular ordinal item triggers the firing of a particular content item, which reveals the state of the connecting plastic synapse. We can see that the sequence of three rotations were successfully stored in the plastic connections. Since we focus on the neuronal architecture for storing sequences here rather than on the aspects of the robotic implementation, we did not quantify precision of the sequence reproduction and only accessed it qualitatively (as can be observed in the accompanying video sequence).

## 4. Discussion

In a proof-of-concept demonstration we have shown how sequences can be stored in a mixed signal analog/digital neuromorphic device with on-chip plasticity (Qiao et al., 2015). We have shown that sequences of different length can be learned and updated and how sequence learning can be driven by sensory input from a silicon retina camera DVS.

Computing architectures in neuromorphic hardware can process events efficiently, in an inherently event-based manner, in real-time, and with ultra-low power consumption (e.g., the ROLLS device used here consumes <4 mW if all its neurons fire at an average frequency of 30 Hz). Of course, on the current prototype stage, neuromorphic hardware requires a regular computer (a credit-card size computer Parallella in our case) to connect the device to the robot and monitor its activity, and an FPGA to be configured efficiently, but in a final embedded application, all computation can happen in the device. The main remaining challenge to achieve this are AER interfaces to different sensors and motors, which are currently being developed (Perez-Pena et al., 2014a, 2015). Power saving is of particular importance in small in agile robots, but even for larger robotic systems, in which at the moment motors dominate the power budget, when (spiking) neural networks are used to process multi-modal sensory information and to control the robot, power consumption of their simulation might become substantial for a real-time application.

First robotic architectures that deploy neuromorphic controllers have been introduced over the last years using analog (Milde et al., 2017a) and digital (Conradt et al., 2015) neuromorphic devices. Compared to the mixed-signal neuromorphic hardware used here, digital realizations of neuromorphic computing offer more flexibility in terms of neuronal and synaptic models at the cost of increased power consumption and device size. Choosing a well-suited neuromorphic system is highly task-dependent and it has been shown that large digital neuromorphic devices (e.g., the SpiNNacker platform, Furber et al., 2012) can also be used to control autonomous mobile robots (Conradt et al., 2015). The presented on-chip sequence learning neuromorphic architecture can be realized both in analog and digital neuromorphic devices.

The main limitation when using analog hardware is the mismatch in device parameters that leads to noisy and unreliable computing elements—neurons and synapses. We showed how the use of population-based representations (“place-code”) and attractor dynamics of neural fields allow to nevertheless produce reliable behavior with these noisy elements, thus unleaching this ultra-low power of analog neuromorphic hardware for practical use.

The proposed neuromorphic architecture presents a crucial building block for complex “cognitive” neuromorphic robotic systems, since sequence learning and sequence generation are key to the most basic robotic tasks, such as map formation (in a simultaneous localization and mapping task) or production of motor sequences. Showing that sequences can be learned, updated, and replayed with flexible timing on a neuromorphic device is thus a crucial stepping stone for neuromorphic cognitive robots and for autonomous learning using plastic on-board synapses that realize a local learning rule.

The particular model for sequence representation, used here (Sandamirskaya and Schöner, 2010a), has several properties, advantageous for a neuromorphic implementation: First, it allows for flexible timing of sequential elements during sequence learning and replay, allowing the sensory input signaling completion of actions to drive sequential transitions. Second, it circumvents problems of some other serial order models that have to do with ordinal vs. chaining representation of serial order (Henson, 1998). Thus, repeated items—both on adjacent or distant positions in a sequence—are not a problem for the model. The length of the sequence can be arbitrary and is limited by the required number of neurons, which grows linearly with the sequence length. A model can be easily extended to represent hierarchical sequences (Duran and Sandamirskaya, 2012), sequences of state coming from different modalities (Sandamirskaya and Schöner, 2010b), or sequences with intrinsic timing of transitions (Duran and Sandamirskaya, 2018). Finally, and most importantly, a sequence here can be learned with a very simple Hebbian learning rule in a fast—one shot—learning process. The sequence can be refined and corrected by further repetitions, but it can be replayed already after a single presentation. Thus, we find this model a promising building block for a wide range of future neuromorphic architectures that require storing sequences of states, e.g., in reinforcement learning, map formation, imitation learning, or human-robot interaction.

## Author contributions

RK and DA were the main driving force in neuromorphic realization and conducting experiments with the robots, NQ and GI provided support with neuromorphic hardware and bias tuning. YS has initiated the project, provided the main idea, and guided its implementation in neuromorphic hardware. All contributed to writing.

### Conflict of interest statement

The authors declare that the research was conducted in the absence of any commercial or financial relationships that could be construed as a potential conflict of interest.

## Footnotes

^1^https://inilabs.com/products/pushbot

^2^We drop 80% of events randomly to further reduce events' flow without loss in performance for the simple vision task used here.

## Appendix

### A.1. on-chip biases to realize a wta on the rolls chip

**Table 1:** Biases of the silicon neuron.

**Parameter**	**Description**	**Value**
IF_RST	The reset threshold current	1 pA
IF_DC	A constant current injected to all neurons	1 pA
IF_BUF	Buffer for oscilloscope	10.9 nA
IF_ATHR	Neuron's adaptive threshold current	1 pA
IF_RFR1	The duration of the neuron's refractory period	1.5 nA
IF_RFR2	The duration of a specified neuron's refractory period	1.5 nA
IF_AHW	The adaptation time constant	1 pA
IIF_AHTAU	Neuron's adaptive Tau	7.4 nA
IIF_TAU2	Time constant for specified neurons	22.6 pA
IF_TAU1	Time constant for all other neurons	24 uA
IF_NMDA	The sensitivity of the neurons	1 pA
IF_CASC	The cascade current	1 pA
IF_THR	The neuron's firing threshold	280 nA

**Table 2:** Biases for the recurrent synaptic connections.

**Parameter**	**Description**	**Value: Exp.1**	**Value: Exp.2**
WHT_STD	Controls the magnitude of short term depression	1 pA	1 pA
PWLK	Controls the pulse width of the synaptic current	448.4 pA	69.7 pA
WHT_INH	Controls the magnitude of the excitatory weight independent when set to value 0	3.8 nA	5.5 nA
WHT_INH0	Controls the magnitude of the inhibitory current component which is injected into the excitatory DPI when set to value 1	8.7 nA	198.4 nA
WHT_INH1	Controls the magnitude of the excitatory current component which is injected into the excitatory DPI when set to value 2	199.4 nA	336.7 nA
WHT_EXC	Controls the magnitude of the excitatory weight independent when set to value 0	110.9 nA	689.7 pA
WHT_EXC0	Controls the magnitude of the excitatory current component which is injected into the excitatory DPI when set to value 1	291.2 nA	3.3 uA
WHT_EXC1	Controls the magnitude of the excitatory current component which is injected into the excitatory DPI when set to value 2	1.4 nA	1.4 nA
DPIE_THR	Controls the threshold of excitatory synapses	33.1 nA	5.38 nA
DPIE_TAU	Controls the time constant of excitatory synapses	402.2 pA	853.7 pA
DPII_TAU	Controls the time constant of inhibitory synapses	1 pA	20.5 pA
DPII_THR	Controls the threshold of inhibitory synapses	262.6 nA	33.0 nA
